# Supplementary material for: Sensitivity to food and cocaine cues are independent traits in a large sample of heterogeneous stock rats
Source: Sci Rep. 2021 Jan 26;11:2223. doi: 10.1038/s41598-020-80798-w (PMC7838206; doi:10.1038/s41598-020-80798-w)
Supplement: Supplementary file 1 — Supplementary Figure 1. [file 41598_2020_80798_MOESM1_ESM.pdf]

Sensitivity to food and cocaine cues are independent traits in a large sample of heterogeneous stock rats.

Christopher P. King[1], Jordan A. Tripi[1], Alesa R. Hughson[2], Aidan P. Horvath[2], Alexander C. Lamparelli[1], Katie L. Holl[3], Apurva Chitre[7], Oksana Polesskaya[7], Keita Ishiwari[4,5], Leah C. Solberg Woods[6], Abraham A. Palmer[7,8], Terry E. Robinson[2], Shelly B. Flagel[9,10], *Paul J. Meyer[1]

[1]Department of Psychology, University at Buffalo, Buffalo, USA.
[2]Department of Psychology, University of Michigan, Ann Arbor, USA.
[3]Department of Pediatrics, Human and Molecular Genetics Center and Children's Research Institute, Medical College of Wisconsin, Milwaukee, USA.
[4]Clinical and Research Institute on Addictions, Buffalo, USA.
[5]Department of Pharmacology and Toxicology, University at Buffalo, Buffalo USA
[6]Department of Internal Medicine, Molecular Medicine, Center on Diabetes, Obesity and Metabolism, Wake Forest School of Medicine, Winston-Salem, USA.
[7]Department of Psychiatry, University of California San Diego, La Jolla, USA.
[8]Institute for Genomic Medicine, University of California San Diego, La Jolla, USA.
[9]Department of Psychiatry, University of Michigan, Ann Arbor, USA.
[10]Michigan Neuroscience Institute, University of Michigan, Ann Arbor, USA.

*Correspondence and requests for additional materials should be addressed to Paul J. Meyer, Behavioral Neuroscience Program, Department of Psychology, The University at Buffalo, Park Hall B72, Buffalo, NY 14260, USA, Tel: + 716 645 0263 (email: pmeyer@buffalo.edu)

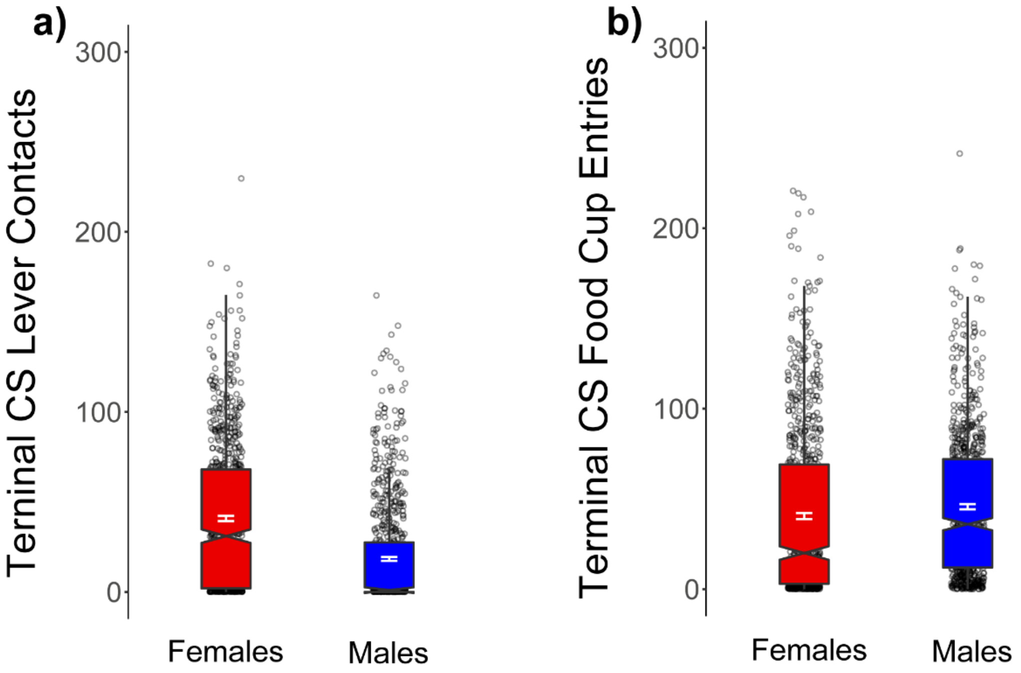

c)

	Change In Time on CS+	Trial 1 Cocaine Dist(mm)	Trial 4 Cocaine Dist(mm)
**Terminal CS Lever Contacts**	*0.005824*	*0.026536*	*0.012502*
**Terminal CS Food Cup Entries**	*0.002600*	*0.004897*	0.000044

Supplementary Figure 1: Terminal lever contacts and food cup entries in males and females and correlations with the measures of conditioned cue preference: A) Males show a positively skewed distribution of lever contacts, but **B)** a more normally distributed number of conditioned food cup entries, consistent with the biased PavCA index distribution towards goal-tracking. Females show only a modest positive skew towards lever contacts and food cup entries, consistent with the more bimodal distribution of sign- and goal-trackers. **C)** The R^2 values for correlations between PavCA responses shown in A) and B), against CCP had very small effect sizes that did not account for much of the variance. Significant correlations are shown in italics.
